# The prevalence and increasing trends of overweight, general obesity, and abdominal obesity among Chinese adults: a repeated cross-sectional study

**DOI:** 10.1186/s12889-019-7633-0

**Published:** 2019-10-15

**Authors:** Yongjie Chen, Qin Peng, Yu Yang, Senshuang Zheng, Yuan Wang, Wenli Lu

**Affiliations:** Department of Epidemiology and Statistics, School of Public Health, Tianjin Medical University, 22 Qixiangtai Road, Tianjin, China

**Keywords:** Body mass index, Waist circumference, General obesity, Abdominal obesity

## Abstract

**Background:**

The prevalence of general and abdominal obesity has increased rapidly in China. The aims of this study were to estimate the dynamic prevalence of overweight, general obesity, and abdominal obesity and the distribution of body mass index (BMI) and waist circumference (WC) among Chinese adults.

**Methods:**

Data were obtained from the China Health and Nutrition Survey (CHNS). According to the suggestions of the WHO for Chinese populations, overweight was defined as a 23 kg/m^2^ ≤ BMI < 27.5 kg/m^2^ and general obesity as a BMI ≥ 27.5 kg/m^2^. Abdominal obesity was defined as a WC ≥ 90 cm for males and ≥ 80 cm for females. Grade 1, grade 2, and grade 3 obesity were defined as 27.5 kg/m^2^ ≤ BMI < 32.5 kg/m^2^, 32.5 kg/m^2^ ≤ BMI < 37.5 kg/m^2^, and BMI ≥ 37.5 kg/m^2^, respectively. Generalized estimation equations were used to estimate the prevalence and trends of overweight, general and abdominal obesity.

**Results:**

This study included 12,543 participant. From 1989 to 2011, the median BMI of males and females increased by 2.65 kg/m^2^ and 1.90 kg/m^2^, respectively; and WC increased by 8.50 cm and 7.00 cm, respectively. In 2011, the age-adjusted prevalence of overweight, general obesity, and abdominal obesity were 38.80% (95% *CI*: 37.95–39.65%), 13.99% (95% *CI*: 13.38–14.59%), and 43.15% (95% *CI*: 42.28–44.01%), respectively, and significantly increased across all cycles of the survey among all subgroups (all *P* < 0.0001). The age-adjusted prevalence of grade 1–3 obesity significantly increased in total sample and sex subgroups (all *P* < 0.0001). For all indicators, there were significant increases in annual *ORs* among all subgroups (all *P* < 0.0001), with the exception of grade 2 obesity. Significant differences were observed in *ORs* across the three age groups in males. And *ORs* significantly decreased with age.

**Conclusions:**

The age-adjusted prevalence of overweight, general obesity, and abdominal obesity significantly increased among Chinese adults from 1989 to 2011. The obesity population is trending toward an increased proportion of males and younger individuals in China.

## Background

Overweight and obesity are important lifestyle-related public health problems worldwide [1, 2]. Since obesity is associated with the common chronic diseases, including cardiovascular disease, type 2 diabetes, hypertension, dyslipidemia, and certain types of cancer, and considered as the fifth leading risk factors for mortality globally [2–8], obesity-related issues have drawn more and more attention from researchers in recent decades. Therefore, it is necessary to investigate and monitor the trends in the prevalence of overweight and obesity to improve awareness and make preventive strategies in the public health field.

In recent years, the prevalence of overweight and obesity has reached epidemic proportions in China [9, 10]. Approximately 20% obesity individuals worldwide are Chinese [11]. The considerable increase in the prevalence of obesity is attributed to the adoption of a Western lifestyle and decreased physical activity [12]. The traditional Chinese diet, characterized by a high carbohydrate content composed of rice, wheat, and cooked vegetables, is shifting to a diet with higher fat [13, 14]. The high intake of energy and fat combined with a decrease in physical activity are responsible for the increasing prevalence of overweight and obesity in the Chinese population, especially among urban inhabitants [15, 16]. Depicting the trends in the prevalence of obesity will help elucidate the prevalence of obesity-related chronic diseases and alert health care professionals and the public to prevent the epidemic.

Body mass index (BMI) is a common indicator used to identify general obesity [9]. Waist circumference (WC) can provide information on the distribution of body fat and is strongly correlated with central fat localization [17–19]. Therefore, BMI and WC were used to define general and abdominal obesity in this study, respectively. Since ethnicities and dietary patterns are different in different countries, the prevalence and extent of obesity vary. Previous studies have reported that Asians have higher body fat content than Western people with the same BMI [20, 21]. Therefore, specific cut-offs of BMI should be used to define overweight and obesity in different countries. In this study, ethnicity-based cut-offs for BMI were used to define overweight and obesity according to the WHO recommendations for Chinese people. Based on the China Health and Nutrition Survey (CHNS), the aims of this study were to investigate the trends in the prevalence of overweight, general obesity, and abdominal obesity as well as the distributions of BMI and WC among the Chinese population. As a result, this study would provide more comprehensive and accurate evidence of the trend and distribution of general and abdominal obesity during the last three decades in China.

## Methods

### Study design

As an ongoing open cohort and international collaborative project between the Carolina Population Center at the University of North Carolina at Chapel Hill and the National Institute for Nutrition and Health (NINH, formerly the National Institute of Nutrition and Food Safety) at the Chinese Center for Disease Control and Prevention (CCDC), the CHNS was designed to examine the effects of the health, nutrition, and family planning policies and programs implemented by national and local governments. Furthermore, how the social and economic transformation of the Chinese society is affecting the health and nutritional status of its population is explored in this survey. Nine provinces varying substantially in geography, economic development, public resources, and health indicators are covered in the CHNS. A multistage, random cluster process was used to obtain the samples in each province. Counties in the nine provinces were stratified by income (low, middle, and high). And a weighted sampling scheme was used to randomly select four counties from each province. In addition, the provincial capital and a lower income city were selected when feasible; however, other large cities rather than provincial capitals had to be selected in two provinces. Villages and townships within the counties and urban/suburban neighborhoods within the cities were selected randomly. The sample is diverse, with variation in a wide-ranging set of socioeconomic factors (income, employment, education, and modernization) and other related health, nutritional, and demographic measures. Because of the long duration and wide geographic coverage, the CHNS can represent the population demographics of China and document the dramatic economic, social, behavioral, and health status changes that have impacted China. The first round of the CHNS was conducted in 1989, and the survey was subsequently conducted in 1991, 1993, 1997, 2000, 2004, 2006, 2009, and 2011. A detailed description of the survey design and procedures has been published elsewhere [22].

### Study population

Data were obtained from all nine waves of the CHNS conducted from 1989 to 2011. The inclusion criteria was as following: those aged ≥18 years at baseline; those with available data on sex and detailed physical examination (e.g., weight and height). The exclusion criteria was as following: those being pregnant or lactating at the time of survey; and those with missing or implausible outlying data (e.g., weight > 300 kg or < 20 kg, WC < 20 cm).

### Measurements and definitions of overweight, general obesity, and abdominal obesity

Weight, height, and WC were measured by trained healthcare workers following standardized protocols and performed at the same location as well as followed the same protocol at each survey visit. Height was measured to the nearest 0.1 cm without wearing shoes using a portable stadiometer. Weight was measured to the nearest 0.1 kg using a calibrated beam scale while wearing lightweight clothing. BMI was calculated as weight (in kg) divided by the square of height (in m). WC was measured at a point midway between the lowest rib and the iliac crest in a horizontal plane using nonelastic tape.

Since the WHO proposed the additional trigger points to define overweight and obesity for public health action in Asian populations, it was more significant to reflect the trends of overweight and obesity according to the suggestions of the WHO for Chinese population [23]. Therefore, overweight was defined as a 23.0 kg/m^2^ ≤ BMI < 27.5 kg/m^2^, and general obesity was defined as a BMI ≥ 27.5 kg/m^2^. Abdominal obesity was defined as a WC ≥ 90 cm for males and ≥ 80 cm for females. Grade 1, grade 2, and grade 3 obesity were defined as 27.5 kg/m^2^ ≤ BMI < 32.5 kg/m^2^, 32.5 kg/m^2^ ≤ BMI < 37.5 kg/m^2^, and BMI ≥ 37.5 kg/m^2^, respectively [23].

### Statistical analysis

Data are reported as the median (interquartile range) for BMI and WC and the frequency and percent (95% confidence interval (*CI*)) for overweight, general obesity, grade 1–3 obesity, and abdominal obesity. Since there was clustering for the subjects from the same household, generalized estimated equations were employed to correct the random effect and analyze the linear trends in the prevalence of overweight, general and abdominal obesity [24, 25]. Analyses were stratified by sex and age, which was defined as 18–39 years, 40–59 years, and ≥ 60 years. Generalized linear mixed models were used to obtain the annual odds ratios (*ORs*) [26]. In this study, the direct method was used to obtain the age-adjusted prevalence of general and abdominal obesity. The data from the Chinese population census in 2010 were considered as the reference. First, the expected number of individuals with obesity was calculated as the prevalence of obesity in each age- subgroup multiplied by the number from the population censuses in the corresponding age- subgroup. Second, the total expected number of individuals with obesity was calculated as the sum of the expected number of obesity individuals in each age- subgroup. Third, the age-adjusted prevalence of obesity was calculated as the total expected number of obesity individuals divided by the total number of individuals from the population census. Similarly, the age-adjusted prevalence of overweight, grade 1–3 obesity, and abdominal obesity were obtained. All analyses were conducted in SAS 9.4 (SAS Institute Inc., Cary, NC, USA). A two-tailed test was used, and the significance level was set at *α* = 0.05.

## Results

The characteristics of the nine waves of the CHNS conducted from 1989 to 2011 are presented in Table 1. The sample sizes of the nine waves were 5080 in 1989, 8382 in 1991, 8017 in 1993, 8473 in 1997, 9374 in 2000, 9100 in 2004, 9039 in 2006, 9426 in 2009, and 12,543 in 2011.

**Table 1:** The characteristics of CHNS from 1989 to 2011

Characteristics	1989	1991	1993	1997	2000	2004	2006	2009	2011
N	5080	8382	8017	8473	9374	9100	9039	9426	12,543
Age									
18–39	4206(82.80)	4395(52.43)	3945(49.21)	3689(43.54)	3773(40.25)	2890(31.76)	2555(28.27)	2425(25.73)	2957(23.57)
40–59	866(17.05)	2718(32.43)	2786(34.75)	3245(38.30)	3807(40.61)	4125(45.33)	4221(46.70)	4391(46.58)	5896(47.01)
60–100	8(0.16)	1269(15.14)	1286(16.04)	1539(18.16)	1794(19.14)	2085(22.91)	2263(25.04)	2610(27.69)	3690(29.42)
Sex									
Males	2401(47.26)	4052(48.34)	3867(48.24)	4171(49.23)	4520(48.22)	4348(47.78)	4255(47.07)	4485(47.58)	5890(46.96)
Females	2679(52.74)	4330(51.66)	4150(51.76)	4302(50.77)	4854(51.78)	4752(52.22)	4784(52.93)	4941(52.42)	6653(53.04)

*CHNS* China Health and Nutrition Survey

The trends in the distributions of BMI and WC from 1989 to 2011 are displayed in Table 2. The median BMI and WC at the follow- up were 23.31 kg/m^2^ and 80 cm, respectively. The median BMI increased significantly from 1989 to 2011 in all subgroups (all *P* < 0.0001). The median BMI increased by 2.65 kg/m^2^ in males and 1.90 kg/m^2^ in females. In the stratified analyses by age, there were linear increasing trends in all subgroups (all *P* < 0.0001), with the exception of the 18–39 years group in women, which did not fall within the linearly increasing trend. The trends in WC were similar with those in BMI. The median WC increased by 8.50 cm in men and 7.00 cm in women. Significant increases in the median WC were observed in all subgroups (all *P* < 0.0001).

**Table 2:** The distribution of body mass index and waist circumference among Chinese adults from the CHNS: 1989–2011

Indicators	1989		1991		1993		1997		2000		2003		2006		2009		2011		*Z*	*P*
	n	M (Q)	n	M (Q)	n	M (Q)	n	M (Q)	n	M (Q)	n	M (Q)	n	M (Q)	n	M (Q)	n	M (Q)		
BMI (kg/m^2^)																				
Total	5080	21.20(3.06)	8382	21.20(3.58)	8017	21.40(3.62)	8473	21.80(4.04)	9374	22.40(4.36)	9100	22.60(4.53)	9039	22.80(4.47)	9426	23.04(4.63)	12,543	23.50(4.74)	61.74	<.0001
Men																				
Overall	2401	21.01(2.83)	4052	21.05(3.26)	3867	21.30(3.26)	4171	21.73(3.77)	4520	22.31(4.26)	4348	22.67(4.36)	4255	22.84(4.43)	4485	23.09(4.53)	5890	23.66(4.59)	47.09	<.0001
Age (years)																				
18–39	1969	20.91(2.73)	2150	20.87(2.90)	1918	21.05(2.93)	1888	21.47(3.34)	1878	21.97(3.90)	1413	22.27(4.13)	1219	22.50(4.19)	1189	22.57(4.86)	1354	23.39(5.15)	33.19	<.0001
40–59	431	21.55(3.28)	1304	21.46(3.54)	1335	21.72(3.50)	1559	22.10(3.93)	1809	22.71(4.21)	1955	23.05(4.21)	1987	23.18(4.18)	2068	23.53(4.35)	2782	24.00(4.33)	26.43	<.0001
60–100	1	22.96(0.00)	598	21.09(4.09)	614	21.21(4.08)	724	21.77(4.74)	833	22.23(4.86)	980	22.44(4.91)	1049	22.49(4.89)	1228	22.80(4.64)	1754	23.24(4.72)	11.12	<.0001
Women																				
Overall	2679	21.48(3.30)	4330	21.44(3.93)	4150	21.58(4.07)	4302	22.02(4.23)	4854	22.61(4.45)	4752	22.74(4.72)	4784	22.82(4.52)	4941	22.99(4.72)	6653	23.38(4.86)	40.47	<.0001
Age (years)																				
18–39	2237	21.37(3.17)	2245	21.14(3.26)	2027	21.19(3.44)	1801	21.47(3.47)	1895	21.76(3.78)	1477	21.69(3.82)	1336	21.64(3.88)	1236	21.55(4.29)	1603	21.72(4.07)	15.49	<.0001
40–59	435	22.07(3.88)	1414	22.04(4.52)	1451	22.31(4.42)	1686	22.73(4.41)	1998	23.48(4.30)	2170	23.42(4.45)	2234	23.41(4.40)	2323	23.61(4.38)	3114	24.03(4.56)	21.03	<.0001
60–100	7	20.08(3.63)	671	21.27(5.05)	672	21.62(5.09)	815	22.03(5.33)	961	22.48(5.22)	1105	22.83(5.22)	1214	23.10(5.08)	1382	23.20(5.15)	1936	23.57(4.98)	11.12	<.0001
WC (cm)																				
Total	–	–	–	–	8017	75.00(11.00)	8473	76.00(12.00)	9374	78.00(14.00)	9100	80.00(14.00)	9039	80.30(14.00)	9426	82.00(15.00)	12,543	83.50(14.80)	70.59	<.0001
Men																				
Overall	–	–	–	–	3867	75.00(12.00)	4171	78.00(13.00)	4520	80.00(13.00)	4348	82.00(14.00)	4255	82.40(14.00)	4485	84.00(14.00)	5890	86.00(13.80)	55.64	<.0001
Age (years)																				
18–39	–	–	–	–	1918	74.00(9.50)	1888	76.00(11.00)	1888	78.00(12.00)	1413	80.00(13.00)	1219	80.50(13.00)	1189	81.50(15.10)	1354	84.00(15.60)	34.70	<.0001
40–59	–	–	–	–	1335	77.00(11.00)	1559	79.00(13.00)	1559	81.00(13.00)	1955	83.00(13.00)	1987	83.60(13.00)	2068	85.00(13.20)	2782	87.00(13.00)	33.55	<.0001
60–100	–	–	–	–	614	78.00(13.00)	724	80.00(16.00)	724	82.00(15.00)	980	82.50(14.90)	1049	83.00(15.00)	1228	84.50(14.60)	1754	86.00(13.90)	15.56	<.0001
Women																				
Overall	–	–	–	–	4150	74.00(12.00)	4302	75.00(12.00)	4854	77.00(14.00)	4752	78.50(14.00)	4784	79.00(13.00)	4941	80.00(14.00)	6653	81.00(14.60)	45.51	<.0001
Age (years)																				
18–39	–	–	–	–	2027	72.00(9.00)	1801	72.00(10.00)	1895	74.00(11.00)	1477	74.00(11.00)	1477	74.00(10.50)	1477	75.00(13.00)	1603	76.00(12.80)	20.57	<.0001
40–59	–	–	–	–	1451	76.00(13.00)	1686	77.00(12.00)	1998	80.00(13.00)	2170	80.00(13.00)	2170	80.00(13.00)	2170	81.00(12.80)	3114	82.00(13.30)	23.96	<.0001
60–100	–	–	–	–	672	78.00(15.00)	815	79.00(16.00)	961	81.00(15.00)	1105	82.00(16.00)	1105	82.00(15.00)	1105	84.00(14.20)	1936	84.50(14.30)	14.42	<.0001

*CHNS* China Health and Nutrition Survey; *BMI* body mass index; *WC* waist circumference

The prevalence of overweight, general obesity, and abdominal obesity are reported in Table 3. In total, the age-adjusted prevalence of overweight increased significantly from 23.82 to 38.80% (*P* < 0.0001). The age- adjusted prevalence of overweight increased significantly from 16.49 to 42.04% in men (*P* < 0.0001) and from 27.44 to 36.06% in women (*P* < 0.0001). Moreover, the prevalence of overweight in men (95% *CI*: 40.78–43.30%) was greater than that in women (95% *CI*: 34.91–37.22%) in 2011. In all age groups, significant increases in the prevalence of overweight were observed in both men and women (*P* < 0.0001). Similarly, the age-adjusted prevalence of general obesity increased from 2.15 to 13.99% in total, from 1.46 to 14.99% in men, and from 2.78 to 13.22% in women (all *P* < 0.0001). There were significant increases in the prevalence of general obesity among all subgroups (all *P* < 0.0001). There were significant increases in the age-adjusted prevalence of abdominal obesity in the total sample (from 19.84 to 43.15%, *P* < 0.0001), in men (from 9.17 to 34.70%, *P* < 0.0001), and in women (from 29.75 to 50.75%, *P* < 0.0001). Compared to men, there was a higher prevalence of abdominal obesity among women across all age groups and cycles of surveys.

**Table 3:** The prevalence of overweight, obesity and abdominal obesity among Chinese adults from the CHNS: 1989–2011

Indicators	1989		1991		1993		1997		2000		2004		2006		2009		2011		*Z*	*P*
	n	%(*CI*)	n	%(*CI*)	n	%(*CI*)	n	%(*CI*)	n	%(*CI*)	n	%(*CI*)	n	%(*CI*)	n	%(*CI*)	n	%(*CI*)		
Overweight																				
Total	1080	21.26 (20.13–22.38)	1983	23.66 (22.75–24.57)	2032	25.35 (24.39–26.30)	2469	29.14 (28.17–30.11)	3270	34.88 (33.92–35.85)	3335	36.65 (35.66–37.64)	3405	37.67 (36.67–38.67)	3654	38.77 (37.78–39.75)	5141	40.99 (40.13–41.85)	32.51	<.0001
Adjusted^a^	1080	23.82 (22.65–25.00)	1983	24.15 (23.23–25.06)	2032	25.64 (24.68–26.59)	2469	28.93 (27.97–29.90)	3270	34.34 (33.38–35.30)	3335	35.38 (34.39–36.36)	3405	36.01 (35.02–37.00)	3654	36.27 (35.30–37.24)	5141	38.80 (37.95–39.65)		
Men																				
Overall	422	17.58 (16.05–19.10)	839	20.71 (19.46–21.95)	885	22.89 (21.56–24.21)	1144	27.43 (26.07–28.78)	1529	33.83 (32.45–35.21)	1607	36.96 (35.52–38.39)	1640	38.54 (37.08–40.01)	1797	40.07 (38.63–41.50)	2535	43.04 (41.77–44.30)	29.03	<.0001
Adjusted^a^	422	16.49 (15.01–17.97)	839	21.19 (19.93–22.45)	885	23.17 (21.84–24.50)	1144	27.38 (26.03–28.74)	1529	33.47 (32.10–34.85)	1607	36.26 (34.83–37.69)	1640	37.59 (36.14–39.05)	1797	38.36 (36.94–39.78)	2535	42.04 (40.78–43.30)		
Age (years)																				
18–39	318	16.15 (14.52–17.78)	376	17.49 (15.88–19.09)	365	19.03 (17.27–20.79)	435	23.04 (21.14–24.94)	546	29.07 (27.02–31.13)	468	33.12 (30.67–35.58)	415	34.04 (31.38–36.70)	395	33.22 (30.54–35.90)	526	38.85 (36.25–41.44)	18.88	<.0001
40–59	104	24.13 (20.09–28.17)	334	25.61 (23.24–27.98)	381	28.54 (26.12–30.96)	514	32.97 (30.64–35.30)	706	39.03 (36.78–41.28)	795	40.66 (38.49–42.84)	852	42.88 (40.70–45.05)	909	43.96 (41.82–46.09)	1298	46.66 (44.80–48.51)	14.74	<.0001
60–100	0	0.00	129	21.57 (18.28–24.87)	139	22.64 (19.33–25.95)	195	26.93 (23.70–30.17)	277	33.25 (30.05–36.45)	344	35.10 (32.11–38.09)	373	35.56 (32.66–38.45)	493	40.15 (37.40–42.89)	711	40.54 (38.24–42.83)	9.80	<.0001
Women																				
Overall	658	24.56 (22.93–26.19)	1144	26.42 (25.11–27.73)	1147	27.64 (26.28–29.00)	1325	30.80 (29.42–32.18)	1741	35.87 (34.52–37.22)	1728	36.36 (35.00–37.73)	1765	36.89 (35.53–38.26)	1857	37.58 (36.23–38.93)	2606	39.17 (38.00–40.34)	17.18	<.0001
Adjusted^a^	658	27.44 (25.75–29.13)	1144	26.90 (25.58–28.22)	1147	27.93 (26.56–29.29)	1325	30.40 (29.03–31.77)	1741	35.10 (33.76–36.45)	1728	34.50 (33.15–35.85)	1765	34.59 (33.24–35.94)	1857	34.27 (32.95–35.59)	2606	36.06 (34.91–37.22)		
Age (years)																				
18–39	515	23.02 (21.28–24.77)	507	22.58 (20.85–24.31)	454	22.40 (20.58–24.21)	446	24.76 (22.77–26.76)	547	28.87 (26.83–30.91)	403	27.29 (25.01–29.56)	364	27.25 (24.86–29.63)	308	24.92 (22.51–27.33)	445	27.76 (25.57–29.95)	6.17	<.0001
40–59	141	32.41 (28.02–36.81)	457	32.32 (29.88–34.76)	502	34.60 (32.15–37.04)	632	37.49 (35.17–39.80)	877	43.89 (41.72–46.07)	948	43.69 (41.60–45.77)	955	42.75 (40.70–44.80)	1037	44.64 (42.62–46.66)	1393	44.73 (42.99–46.48)	8.83	<.0001
60–100	2	28.57 (0.00–62.04)	180	26.83 (23.47–30.18)	191	28.42 (25.01–31.83)	247	30.31 (27.15–33.46)	317	32.99 (30.01–35.96)	377	34.12 (31.32–36.91)	446	36.74(34.03–39.45)	512	37.05 (34.50–39.59)	768	39.67 (37.49–41.85)	5.90	<.0001
Obesity																				
Total	100	1.97 (1.59–2.35)	331	3.95 (3.53–4.37)	333	4.15 (3.72–4.59)	553	6.53 (6.00–7.05)	803	8.57 (8.00–9.13)	901	9.90 (9.29–10.51)	940	10.40 (9.77–11.03)	1102	11.69 (11.04–12.34)	1855	14.79 (14.17–15.41)	32.27	<.0001
Adjusted^a^	100	2.15 (1.75–2.54)	331	4.24 (3.81–4.67)	333	4.26 (3.82–4.71)	553	6.41 (5.89–6.93)	803	8.31 (7.76–8.87)	901	9.20 (8.61–9.79)	940	9.69 (9.08–10.30)	1102	11.02 (10.39–11.65)	1855	13.99 (13.38–14.59)		
Men																				
Overall	30	1.25 (0.81–1.69)	125	3.08 (2.55–3.62)	119	3.08 (2.53–3.62)	238	5.71 (5.00–6.41)	334	7.39 (6.63–8.15)	389	8.95 (8.10–9.80)	401	9.42 (8.55–10.30)	495	11.04 (10.12–11.95)	853	14.48 (13.58–15.38)	24.18	<.0001
Adjusted^a^	30	1.46 (0.98–1.94)	125	3.30 (2.75–3.85)	119	3.15 (2.59–3.70)	238	5.65 (4.95–6.35)	334	7.32 (6.56–8.08)	389	8.60 (7.76–9.43)	401	9.53 (8.64–10.41)	495	11.44 (10.51–12.37)	853	14.99(14.08–15.90)		
Age (years)																				
18–39	18	0.91 (0.49–1.33)	33	1.53 (1.02–2.05)	36	1.88 (1.27–2.48)	77	4.08 (3.19–4.97)	124	6.60 (5.48–7.73)	101	7.15 (5.80–8.49)	118	9.68 (8.02–11.34)	142	11.94 (10.10–13.79)	212	15.66 (13.72–17.59)	20.55	<.0001
40–59	12	2.78 (1.23–4.34)	55	4.22 (3.13–5.31)	49	3.67 (2.66–4.68)	94	6.03 (4.85–7.21)	138	7.63 (6.41–8.85)	199	10.18 (8.84–11.52)	197	9.91 (8.60–11.23)	255	12.33 (10.91–13.75)	430	15.46 (14.11–16.80)	13.19	<.0001
60–100	0	0.00	37	6.19 (4.26–8.12)	34	5.54 (3.73–7.35)	67	9.25(7.14–11.37)	72	8.64 (6.74–10.55)	89	9.08 (7.28–10.88)	86	8.20 (6.54–9.86)	98	7.98 (6.46–9.50)	211	12.03(10.51–13.55)	4.03	<.0001
Women																				
Overall	70	2.61(2.01–3.22)	206	4.76(4.12–5.39)	214	5.16 (4.48–5.83)	315	7.32 (6.54–8.10)	469	9.66 (8.83–10.49)	512	10.77 (9.89–11.66)	539	11.27 (10.37–12.16)	607	12.28 (11.37–13.20)	1002	15.06 (14.20–15.92)	21.38	<.0001
Adjusted^a^	70	2.78 (2.16–3.40)	206	5.10 (4.45–5.76)	214	5.30 (4.62–5.98)	315	7.11 (6.34–7.87)	469	9.19 (8.38–10.00)	512	9.75 (8.90–10.59)	539	9.83 (8.98–10.67)	607	10.60 (9.74–11.46)	1002	13.22 (12.40–14.03)		
Age (years)																				
18–39	49	2.19 (1.58–2.80)	44	1.96 (1.39–2.53)	55	2.71 (2.01–3.42)	77	4.28 (3.34–5.21)	104	5.49 (4.46–6.51)	90	6.09 (4.87–7.31)	75	5.61 (4.38–6.85)	79	6.39 (5.03–7.76)	138	8.61 (7.24–9.98)	11.49	<.0001
40–59	21	4.83 (2.81–6.84)	111	7.85 (6.45–9.25)	105	7.24 (5.90–8.57)	152	9.02 (7.65–10.38)	238	11.91 (10.49–13.33)	263	12.12 (10.75–13.49)	286	12.80 (11.42–14.19)	317	13.65 (12.25–15.04)	536	17.21 (15.89–18.54)	10.92	<.0001
60–100	0	0.00	51	7.60 (5.60–9.61)	54	8.04 (5.98–10.09)	86	10.55 (8.44–12.66)	127	13.22 (11.07–15.36)	159	14.39 (12.32–16.46)	178	14.66 (12.67–16.65)	211	15.27 (13.37–17.16)	328	16.94 (15.27–18.61)	7.06	<.0001
Abdominal obesity																				
Total	–	–	–	–	1477	19.33 (18.44–20.22)	1982	24.05 (23.13–24.97)	2901	31.36 (30.41–32.30)	3200	35.67 (34.68–36.66)	3350	37.86 (36.85–38.87)	3994	42.82 (41.81–43.82)	5933	47.34 (46.47–48.22)	51.31	<.0001
Adjusted^a^	–	–	–	–	1477	19.84 (18.96–20.71)	1982	23.56 (22.65–24.46)	2901	30.18 (29.25–31.11)	3200	32.73 (31.76–33.69)	3350	34.41 (33.43–35.39)	3994	38.68 (37.7–39.66)	5933	43.15 (42.28–44.01)		
Men																				
Overall	–	–	–	–	330	8.96 (8.04–9.88)	595	14.64 (13.55–15.73)	921	20.63 (19.44–21.81)	1017	23.75 (22.47–25.02)	1064	25.53 (24.21–26.86)	1344	30.30 (28.95–31.66)	2139	36.34 (35.11–37.57)	35.57	<.0001
Adjusted^a^	–	–	–	–	330	9.17 (8.26–10.08)	595	14.49 (13.42–15.56)	921	20.20 (19.03–21.37)	1017	22.43 (21.19–23.67)	1064	24.06 (22.77–25.34)	1344	28.61 (27.29–29.94)	2139	34.70 (33.49–35.92)		
Age (years)																				
18–39	–	–	–	–	92	5.04 (4.04–6.05)	181	9.85 (8.49–11.22)	295	15.91 (14.25–17.58)	242	17.39 (15.39–19.38)	233	19.55 (17.30–21.80)	281	23.94 (21.49–26.38)	414	30.60 (28.14–33.05)	21.60	<.0001
40–59	–	–	–	–	141	11.09 (9.37–12.82)	246	16.19 (14.34–18.05)	395	22.14 (20.21–24.07)	509	26.44 (24.47–28.41)	546	28.04 (26.05–30.04)	674	32.88 (30.84–34.91)	1083	38.96 (37.14–40.77)	21.14	<.0001
60–100	–	–	–	–	97	16.47 (13.47–19.46)	168	23.73 (20.06–26.86)	231	27.93 (24.87–30.99)	266	27.54 (24.72–30.35)	285	27.72 (24.99–30.46)	389	32.12 (29.49–34.75)	642	36.62 (34.37–38.88)	10.21	<.0001
Women																				
Overall	–	–	–	–	1147	28.99 (27.57–30.40)	1387	33.21 (31.78–34.63)	1980	41.36 (39.97–42.76)	2183	46.57 (45.14–47.99)	2286	48.83 (47.39–50.26)	2650	54.16 (52.76–55.56)	3794	57.09 (55.90–58.28)	37.81	<.0001
Adjusted^a^	–	–	–	–	1147	29.75 (28.36–31.14)	1387	32.18 (30.78–33.57)	1980	39.32 (37.95–40.70)	2183	42.13 (40.73–43.54)	2286	43.66 (42.26–45.07)	2650	47.85 (46.46–49.24)	3794	50.75 (49.55–51.95)		
Age (years)																				
18–39	–	–	–	–	308	15.97 (14.33–17.60)	333	19.09 (17.25–20.94)	442	23.76 (21.83–25.70)	381	26.13 (23.88–28.39)	373	28.56 (26.11–31.01)	395	32.30 (29.68–34.92)	574	35.85 (33.50–38.20)	16.19	<.0001
40–59	–	–	–	–	540	38.71 (36.15–41.27)	674	41.22 (38.84–43.61)	1003	50.76 (48.55–52.96)	1157	53.89 (51.78–56.00)	1193	54.50 (52.41–56.59)	1348	58.53 (56.52–60.54)	1901	61.11 (59.39–62.82)	18.03	<.0001
60–100	–	–	–	–	299	47.24 (43.35–51.12)	380	47.62 (44.15–51.08)	535	56.26 (53.10–59.41)	645	59.56 (56.63–62.48)	720	60.66 (57.88–63.44)	907	66.35 (63.84–68.85)	1319	68.20 (66.13–70.28)	12.05	<.0001

^a^Adjusted by the direct method to the year 2010 Census population using the age groups 18–39 years, 40–59 years, and 60–100 years

*CHNS* China Health and Nutrition Survey

Table 4 shows the prevalence of overweight, general obesity, and abdominal obesity in different smoking status, marital status, and educational levels. In all subgroups, the prevalence of the three indicators increased significantly, with the exception of overweight in the divorced group (*P* = 0.2193). The higher prevalence of overweight, general obesity, and abdominal obesity were found in non- smoking group. The higher prevalence of abdominal obesity was found in the widowed group and the group with a primary education or no degree.

**Table 4:** The prevalence of overweight, obesity and abdominal obesity among Chinese adults in different smoking status, married status, and education degree from the CHNS: 1989–2011

Indicators	1989		1991		1993		1997		2000		2004		2006		2009		2011		*Z*	*P*
	n	%(*CI*)	n	%(*CI*)	n	%(*CI*)	n	%(*CI*)	n	%(*CI*)	n	%(*CI*)	n	%(*CI*)	n	%(*CI*)	n	%(*CI*)		
Overweight																				
Smoking status																				
No smoking	–	–	1371	25.61 (24.44–26.78)	1407	27.04 (25.84–28.25)	1733	30.57 (29.37–31.77)	2261	35.67 (34.49–36.85)	2274	37.04 (35.83–38.24)	2359	37.94 (36.74–39.15)	2485	38.42 (37.23–39.61)	3575	41.13 (40.10–42.17)	21.23	<.0001
Smoking	–	–	603	20.11 (18.67–21.54)	612	22.34 (20.78–23.90)	718	26.19 (24.54–27.83)	982	33.20 (31.50–34.90)	1056	35.82 (34.09–37.55)	1046	37.07 (35.28–38.85)	1169	39.52 (37.76–41.28)	1565	40.64 (39.09–42.19)	20.99	<.0001
Married status																				
Never married	89	11.38 (9.16–13.61)	173	13.32 (11.47–15.17)	169	14.02 (12.06–15.99)	213	18.08 (15.88–20.28)	242	21.67 (19.25–24.08)	189	23.68 (20.73–26.63)	154	24.25 (20.92–27.59)	107	17.86 (14.8–20.93)	169	24.28 (21.1–27.47)	9.43	<.0001
Married	982	23.07 (21.81–24.34)	1697	26.15 (25.08–27.22)	1738	27.98 (26.87–29.10)	2110	31.94 (30.81–33.06)	2714	37.52 (36.40–38.63)	2873	38.54 (37.44–39.65)	2964	39.21 (38.11–40.31)	3206	40.72 (39.64–41.81)	4505	42.80 (41.85–43.74)	26.59	<.0001
Divorced	4	21.05 (2.72–39.38)	18	30.00 (18.40–41.60)	15	35.71 (21.22–50.21)	14	20.90 (11.16–30.63)	28	30.77 (21.29–40.25)	51	43.59 (34.60–52.57)	43	36.13 (27.50–44.77)	62	37.58 (30.19–44.97)	93	32.98 (27.49–38.47)	1.23	0.2193
Widowed	5	23.81 (5.59–42.03)	93	18.24 (14.88–21.59)	100	19.88 (16.39–23.37)	123	22.49 (18.99–25.98)	158	27.67 (24–31.34)	203	29.99 (26.53–33.44)	235	33.86 (30.34–37.38)	267	35.74 (32.31–39.18)	346	35.67 (32.66–38.68)	8.22	<.0001
Education degree																				
Primary school or none	509	21.23 (19.59–22.86)	1184	24.74 (23.52–25.97)	1115	26.03 (24.71–27.34)	1225	28.84 (27.48–30.21)	1433	34.66 (33.21–36.11)	1432	35.60 (34.12–37.07)	1414	36.59 (35.08–38.11)	1489	37.30 (35.80–38.80)	1756	38.62 (37.20–40.03)	19.95	<.0001
Middle school degree	533	21.34 (19.73–22.94)	741	21.93 (20.53–23.32)	844	24.43 (23.00–25.86)	1098	29.23 (27.78–30.69)	1589	34.98 (33.59–36.36)	1761	37.54 (36.15–38.93)	1788	38.44 (37.04–39.83)	1960	39.89 (38.52–41.26)	2754	42.82 (41.61–44.03)	23.77	<.0001
College or above	22	19.64 (12.28–27.00)	54	30.86 (24.01–37.70)	47	35.34 (27.21–43.46)	76	37.07 (30.46–43.68)	142	37.27 (32.42–42.13)	136	37.06 (32.12–42.00)	198	39.76 (35.46–44.06)	201	39.64 (35.39–43.90)	623	40.43 (37.98–42.88)	4.52	<.0001
Obesity																				
Smoking status																				
No smoking	–	–	243	4.54 (3.98–5.10)	238	4.57 (4.01–5.14)	410	7.23 (6.56–7.91)	587	9.26 (8.55–9.97)	657	10.70 (9.93–11.47)	687	11.05 (10.27–11.83)	812	12.55 (11.75–13.36)	1315	15.13 (14.38–15.88)	23.48	<.0001
Smoking	–	–	88	2.93 (2.33–3.54)	86	3.14 (2.49–3.79)	137	5.00 (4.18–5.81)	209	7.07 (6.14–7.99)	242	8.21 (7.22–9.20)	253	8.97 (7.91–10.02)	290	9.80 (8.73–10.88)	540	14.02 (12.93–15.12)	18.31	<.0001
Married status																				
Never married	3	0.38 (0.00–0.82)	8	0.62 (0.19–1.04)	9	0.75 (0.26–1.23)	20	1.70 (0.96–2.44)	42	3.76 (2.64–4.88)	38	4.76 (3.28–6.24)	23	3.62 (2.17–5.08)	34	5.68 (3.82–7.53)	68	9.77 (7.56–11.98)	12.22	<.0001
Married	94	2.21 (1.77–2.65)	293	4.51 (4.01–5.02)	294	4.73 (4.21–5.26)	483	7.31 (6.68–7.94)	680	9.40 (8.73–10.07)	779	10.45 (9.76–11.15)	833	11.02 (10.31–11.73)	958	12.17 (11.45–12.89)	1594	15.14 (14.46–15.83)	26.58	<.0001
Divorced	2	10.53 (0.00–24.33)	4	6.67 (0.35–12.98)	1	2.38 (0.00–6.99)	5	7.46 (1.17–13.76)	4	4.40 (0.18–8.61)	7	5.98 (1.69–10.28)	6	5.04 (1.11–8.97)	14	8.48 (4.23–12.74)	36	12.77 (8.87–16.66)	2.10	0.0354
Widowed	1	4.76 (0.00–13.87)	26	5.10 (3.19–7.01)	25	4.97 (3.07–6.87)	41	7.50 (5.29–9.70)	51	8.93 (6.59–11.27)	72	10.64 (8.31–12.96)	76	10.95 (8.63–13.27)	88	11.78 (9.47–14.09)	154	15.88 (13.58–18.18)	6.96	<.0001
Education degree																				
Primary school or none	56	2.34 (1.73–2.94)	230	4.81 (4.20–5.41)	210	4.90 (4.26–5.55)	294	6.92 (6.16–7.69)	356	8.61 (7.76–9.47)	417	10.37 (9.42–11.31)	429	11.10 (10.11–12.09)	510	12.78 (11.74–13.81)	688	15.13 (14.09–16.17)	20.54	<.0001
Middle school degree	41	1.64 (1.14–2.14)	91	2.69 (2.15–3.24)	108	3.13 (2.55–3.71)	218	5.80 (5.06–6.55)	371	8.17 (7.37–8.96)	439	9.36 (8.52–10.19)	468	10.06 (9.20–10.92)	539	10.97 (10.10–11.84)	968	15.05 (14.18–15.92)	23.17	<.0001
College or above	2	1.79 (0.00–4.24)	7	4.00 (1.10–6.90)	4	3.01 (0.10–5.91)	18	8.78 (4.91–12.65)	36	9.45 (6.51–12.39)	44	11.99 (8.67–15.31)	41	8.23 (5.82–10.65)	52	10.26 (7.62–12.9)	196	12.72 (11.06–14.38)	4.66	<.0001
Abdominal obesity																				
Smoking status																				
No smoking	–	–	–	–	1194	24.08 (22.89–25.27)	1578	28.65 (27.46–29.84)	2252	36.00 (34.81–37.19)	2491	41.13 (39.89–42.37)	2625	43.19 (41.94–44.43)	3115	48.66 (47.44–49.89)	4494	51.74 (50.69–52.79)	39.93	<.0001
Smoking	–	–	–	–	263	10.06 (8.91–11.21)	391	14.58 (13.25–15.92)	620	21.24 (19.76–22.72)	705	24.29 (22.73–25.85)	725	26.16 (24.53–27.80)	879	30.03 (28.37–31.69)	1439	37.42 (35.89–38.94)	28.06	<.0001
Married status																				
Never married	–	–	–	–	71	6.18 (4.79–7.58)	98	8.54 (6.93–10.16)	153	13.97 (11.92–16.03)	130	16.65 (14.03–19.26)	98	15.68 (12.83–18.53)	94	15.91 (12.96–18.85)	160	23.02 (19.89–26.15)	11.85	<.0001
Married	–	–	–	–	1247	21.04 (20.00–22.07)	1691	26.29 (25.22–27.37)	2397	33.52 (32.42–34.61)	2713	36.87 (35.77–37.97)	2874	38.84 (37.73–39.95)	3415	43.81 (42.71–44.91)	5094	48.42 (47.46–49.37)	42.19	<.0001
Divorced	–	–	–	–	13	33.33 (18.54–48.13)	14	21.54 (11.54–31.53)	18	20.00 (11.74–28.26)	39	33.91 (25.26–42.57)	35	30.17 (21.82–38.53)	55	33.54 (26.31–40.76)	106	37.86 (32.18–43.54)	2.82	0.0049
Widowed	–	–	–	–	138	29.36 (25.24–33.48)	167	31.69 (27.72–35.66)	233	41.31 (37.25–45.38)	301	45.40 (41.61–49.19)	336	49.56 (45.79–53.32)	411	55.77 (52.18–59.35)	548	56.61 (53.49–59.73)	11.95	<.0001
Education degree																				
Primary school or none	–	–	–	–	971	23.86 (22.55–25.17)	1150	27.90 (26.53–29.27)	1449	35.45 (33.98–36.91)	1615	40.73 (39.20–42.26)	1615	42.79 (41.21–44.37)	1935	48.95 (47.39–50.51)	2345	51.63 (50.18–53.08)	32.90	<.0001
Middle school degree	–	–	–	–	447	13.54 (12.37–14.71)	695	19.00 (17.73–20.28)	1210	27.02 (25.72–28.32)	1457	31.52 (30.18–32.86)	1578	34.57 (33.19–35.95)	1879	38.67 (37.30–40.04)	2961	46.06 (44.85–47.28)	37.90	<.0001
College or above	–	–	–	–	31	23.48 (16.25–30.72)	59	28.92 (22.70–35.14)	108	28.88 (24.28–33.47)	123	33.79 (28.93–38.65)	147	30.31 (26.22–34.40)	178	35.46 (31.27–39.64)	616	40.00 (37.55–42.45)	5.47	<.0001

*CHNS* China Health and Nutrition Survey

The prevalence of grade 1, grade 2, and grade 3 combined obesity are presented in Table 5. The age-adjusted prevalence of grade 1 obesity increased significantly in the total sample (from 2.08 to 12.01%, *P* < 0.0001), in men (from 1.38 to 13.25%, *P* < 0.0001), and in women (from 2.74 to 11.03%, *P* < 0.0001). In all age groups, the prevalence of grade 1 obesity increased significantly. Similar trends in the age-adjusted prevalence of grade 2 obesity and grade 3 obesity combined were observed in the total sample as well as both men and women. There were significant increases in the prevalence of grade 2 obesity and grade 3 obesity combined in all age groups except the prevalence of grade 2 obesity in the 60–100 years group (*P* = 0.0629 in men and 0.2130 in women).

**Table 5:** The prevalence of grade 1, grade 2, and grade 2 and grade 3 combined among the Chinese adults from the CHNS: 1989–2011

Indicators	1989		1991		1993		1997		2000		2004		2006		2009		2011		*Z*	*P*
	n	%(*CI*)	n	%(*CI*)	n	%(*CI*)	n	%(*CI*)	n	%(*CI*)	n	%(*CI*)	n	%(*CI*)	n	%(*CI*)	n	%(*CI*)		
Grade 1																				
Total	97	1.91 (1.30–2.90)	308	3.67 (3.27–4.08)	308	3.84 (3.42–4.26)	516	6.09 (5.58–6.60)	739	7.88 (7.34–8.43)	825	9.07 (8.48–9.66)	858	9.49 (8.89–10.10)	1009	10.70 (10.08–11.33)	1599	12.75 (12.16–13.33)	29.02	<.0001
Adjusted^a^	97	2.08 (1.69–2.47)	308	3.93 (3.51–4.34)	308	3.94 (3.51–4.36)	516	5.99 (5.48–6.49)	739	7.66 (7.13–8.20)	825	8.40 (7.83–8.97)	858	8.81 (8.23–9.40)	1009	10.09 (9.48–10.70)	1599	12.01 (11.44–12.58)		
Men																				
Overall	29	1.21 (0.77–1.64)	123	3.04 (2.51–3.56)	114	2.95 (2.41–3.48)	226	5.42 (4.73–6.11)	310	6.86 (6.12–7.60)	368	8.46 (7.64–9.29)	377	8.86 (8.01–9.71)	456	10.17 (9.28–11.05)	751	12.75 (11.90–13.60)	22.09	<.0001
Adjusted^a^	29	1.38 (0.91–1.84)	123	3.24 (2.70–3.79)	114	3.01 (2.47–3.55)	226	5.37 (4.69–6.06)	310	6.81 (6.08–7.55)	368	8.10 (7.29–8.91)	377	8.90 (8.04–9.75)	456	10.58 (9.68–11.48)	751	13.25 (12.38–14.11)		
Age (years)																				
18–39	18	0.91 (0.49–1.33)	33	1.53 (1.02–2.05)	35	1.82 (1.23–2.42)	76	4.03 (3.14–4.91)	119	6.34 (5.23–7.44)	93	6.58 (5.29–7.87)	108	8.86 (7.26–10.45)	132	11.10 (9.32–12.89)	189	13.96 (12.11–15.80)	19.17	<.0001
40–59	11	2.55 (1.06–4.04)	55	4.22 (3.13–5.31)	46	3.45 (2.47–4.42)	90	5.77 (4.62–6.93)	127	7.02 (5.84–8.20)	191	9.77 (8.45–11.09)	188	9.46 (8.17–10.75)	237	11.46 (10.09–12.83)	377	13.55 (12.28–14.82)	11.87	<.0001
60–100	0	0.00	35	5.85 (3.97–7.73)	33	5.37 (3.59–7.16)	60	8.29 (6.28–10.30)	64	7.68 (5.87–9.49)	84	8.57 (6.82–10.32)	81	7.72 (6.11–9.34)	87	7.08 (5.65–8.52)	185	10.55 (9.11–11.98)	3.340	0.0008
Women																				
Overall	68	2.54 (1.94–3.13)	185	4.27 (3.67–4.87)	194	4.67 (4.03–5.32)	290	6.74 (5.99–7.49)	429	8.84 (8.04–9.64)	457	9.62 (8.78–10.46)	481	10.05 (9.20–10.91)	553	11.19 (10.31–12.07)	848	12.75 (11.94–13.55)	19.00	<.0001
Adjusted^a^	68	2.74 (2.12–3.36)	185	4.56 (3.94–5.18)	194	4.79 (4.14–5.44)	290	6.55 (5.81–7.28)	429	8.41 (7.63–9.19)	457	8.67 (7.87–9.47)	481	8.73 (7.93–9.53)	553	9.60 (8.78–10.42)	848	11.03 (10.27–11.78)		
Age (years)																				
18–39	47	2.10 (1.51–2.70)	44	1.96 (1.39–2.53)	55	2.71 (2.01–3.42)	71	3.94 (3.04–4.84)	95	5.01 (4.03–6.00)	78	5.28 (4.14–6.42)	64	4.79 (3.65–5.94)	69	5.58 (4.30–6.86)	108	6.74 (5.51–7.96)	9.24	<.0001
40–59	21	4.83 (2.81–6.84)	98	6.93 (5.61–8.25)	93	6.41 (5.15–7.67)	144	8.54 (7.21–9.88)	223	11.16 (9.78–12.54)	242	11.15 (9.83–12.48)	265	11.86 (10.52–13.20)	295	12.70 (11.35–14.05)	458	14.71 (13.46–15.95)	9.60	<.0001
60–100	0	0.00	43	6.41 (4.56–8.26)	46	6.85 (4.94–8.75)	75	9.2 (7.22–11.19)	111	11.55 (9.53–13.57)	137	12.40 (10.46–14.34)	152	12.52 (10.66–14.38)	189	13.68 (11.86–15.49)	282	14.57 (12.99–16.14)	6.52	<.0001
Grade 2																				
Total	3	0.06 (0.00–0.13)	21	0.25 (0. 14–0.36)	23	0.29 (0. 17–0.40)	34	0.40 (0. 27–0.54)	60	0.64 (0. 48–0.80)	69	0.76 (0. 58–0.94)	75	0.83 (0. 64–1.02)	88	0.93 (0. 74–1.13)	163	1.30 (1.10–1.50)	10.07	<.0001
Adjusted^a^	3	0.06 (0.01–0.13)	21	0.28 (0.17–0.40)	23	0.30 (0.18–0.42)	34	0.39 (0.26–0.52)	60	0.61 (0.45–0.77)	69	0.72 (0.54–0.89)	75	0.81 (0.62–0.99)	88	0.88 (0.69–1.07)	163	1.24 (1.05–1.43)		
Men																				
Overall	1	0.04 (0.00–0.12)	1	0.02 (0.00–0.07)	4	0.10 (0.00–0.20)	12	0.29 (0.13–0.45)	23	0.51 (0.30–0.72)	21	0.48 (0.28–0.69)	22	0.52 (0.30–0.73)	38	0.85 (0.58–1.12)	58	0.98 (0.73–1.24)	7.40	<.0001
Adjusted^a^	1	0.09 (0.00–0.20)	1	0.03 (0.00–0.08)	4	0.11 (0.00–0.21)	12	0.28 (0.12–0.44)	23	0.49 (0.28–0.69)	21	0.50 (0.29–0.71)	22	0.57 (0.35–0.80)	38	0.84 (0.58–1.11)	58	1.03 (0.77–1.29)		
Age (years)																				
18–39	0	0.00	0	0.00	1	0.05 (0.00–0.15)	1	0.05 (0.00–0.16)	5	0.27 (0.03–0.50)	8	0.57 (0.17–0.96)	9	0.74 (0.26–1.22)	10	0.84 (0.32–1.36)	15	1.11 (0.55–1.67)	6.10	<.0001
40–59	1	0.23 (0.00–0.69)	0	0.00	3	0.22 (0.00–0.48)	4	0.26 (0.01–0.51)	11	0.61 (0.25–0.97)	8	0.41 (0.13–0.69)	9	0.45 (0.16–0.75)	17	0.82 (0.43–1.21)	29	1.04 (0.67–1.42)	4.51	<.0001
60–100	0	0.00	1	0.17 (0.00–0.49)	0	0.00	7	0.97 (0.25–1.68)	7	0.84 (0.22–1.46)	5	0.51 (0.06–0.96)	4	0.38 (0.01–0.75)	11	0.90 (0.37–1.42)	14	0.80 (0.38–1.21)	1.86	0.0629
Women																				
Overall	2	0.07 (0.00–0.18)	20	0.46 (0.26–0.66)	19	0.46 (0.25–0.66)	22	0.51 (0.30–0.72)	37	0.76 (0.52–1.01)	48	1.01 (0.73–1.29)	53	1.11 (0.81–1.40)	50	1.01 (0.73–1.29)	105	1.58 (1.28–1.88)	6.94	<.0001
Adjusted^a^	2	0.04 (0.00–0.12)	20	0.52 (0.30–0.73)	19	0.49 (0.27–0.70)	22	0.50 (0.29–0.71)	37	0.72 (0.48–0.96)	48	0.92 (0.65–1.19)	53	1.02 (0.73–1.30)	50	0.92 (0.65–1.19)	105	1.43 (1.14–1.71)		
Age (years)																				
18–39	2	0.09 (0.00–0.21)	0	0.00	0	0.00	6	0.33 (0.07–0.6)	8	0.42 (0.13–0.71)	9	0.61 (0.21–1.01)	11	0.82 (0.34–1.31)	9	0.73 (0.25–1.20)	17	1.06 (0.56–1.56)	6.22	<.0001
40–59	0	0.00	12	0.85 (0.37–1.33)	11	0.76 (0.31–1.20)	7	0.42 (0.11–0.72)	15	0.75 (0.37–1.13)	20	0.92 (0.52–1.32)	18	0.81 (0.44–1.18)	22	0.95 (0.55–1.34)	54	1.73 (1.28–2.19)	3.33	0.0009
60–100	0	0.00	8	1.19 (0.37–2.01)	8	1.19 (0.37–2.01)	9	1.10 (0.39–1.82)	14	1.46 (0.70–2.21)	19	1.72 (0.95–2.49)	24	1.98 (1.19–2.76)	19	1.37 (0.76–1.99)	34	1.76 (1.17–2.34)	1.25	0.2130
Grade 2 and 3																				
Total	3	0.06 (0.00–0.13)	23	0.27 (0.16–0.39)	25	0.31 (0.19–0.43)	37	0.44 (0.30–0.58)	64	0.68 (0.52–0.85)	76	0.84 (0.65–1.02)	82	0.91 (0.71–1.10)	93	0.99 (0.79–1.19)	256	2.04 (1.79–2.29)	12.59	<.0001
Adjusted^a^	3	0.06 (0.00–0.13)	23	0.31 (0.19–0.43)	25	0.33 (0.20–0.45)	37	0.42 (0.28–0.56)	64	0.65 (0.49–0.81)	76	0.80 (0.61–0.98)	82	0.87 (0.68–1.06)	93	0.93 (0.74–1.12)	256	1.98 (1.73–2.22)		
Men																				
Overall	1	0.04 (0.00–0.12)	2	0.05 (0.00–0.12)	5	0.13 (0.02–0.24)	12	0.29 (0.13–0.45)	24	0.53 (0.32–0.74)	21	0.48 (0.28–0.69)	24	0.56 (0.34–0.79)	39	0.87 (0.60–1.14)	102	1.73 (1.40–2.06)	9.63	<.0001
Adjusted^a^	1	0.09 (0.00–0.20)	2	0.05 (0.00–0.13)	5	0.13 (0.02–0.25)	12	0.28 (0.12–0.44)	24	0.51 (0.30–0.71)	21	0.50 (0.29–0.71)	24	0.63 (0.39–0.86)	39	0.86 (0.59–1.13)	102	1.74 (1.41–2.07)		
Ag e (years)																				
18–39	0	0.00	0	0.00	1	0.05 (0.00–0.15)	1	0.05 (0.00–0.16)	5	0.27 (0.03–0.50)	8	0.57 (0.17–0.96)	10	0.82 (0.31–1.33)	10	0.84 (0.32–1.36)	23	1.70 (1.01–2.39)	6.45	<.0001
40–59	1	0.23 (0.00–0.69)	0	0.00	3	0.22 (0.00–0.48)	4	0.26 (0.01–0.51)	11	0.61 (0.25–0.97)	8	0.41 (0.13–0.69)	9	0.45 (0.16–0.75)	18	0.87 (0.47–1.27)	53	1.91 (1.40–2.41)	5.80	<.0001
60–100	0	0.00	2	0.33 (0.00–0.80)	1	0.16 (0.00–0.48)	7	0.97 (0.25–1.68)	8	0.96 (0.30–1.62)	5	0.51 (0.06–0.96)	5	0.48 (0.06–0.89)	11	0.90 (0.37–1.42)	26	1.48 (0.92–2.05)	2.46	0.0139
Women																				
Overall	2	0.07 (0.00–0.18)	21	0.48 (0.28–0.69)	20	0.48 (0.27–0.69)	25	0.58 (0.35–0.81)	40	0.82 (0.57–1.08)	55	1.16 (0.85–1.46)	58	1.21 (0.90–1.52)	54	1.09 (0.80–1.38)	154	2.31 (1.95–2.68)	8.84	<.0001
Adjusted^a^	2	0.04 (0.00–0.12)	21	0.54 (0.33–0.76)	20	0.51 (0.29–0.73)	25	0.56 (0.34–0.78)	40	0.78 (0.53–1.03)	55	1.07 (0.78–1.37)	58	1.10 (0.80–1.39)	54	1.00 (0.72–1.27)	154	2.19 (1.84–2.54)		
Age (years)																				
18–39	2	0.09 (0.00–0.21)	0	0.00	0	0.00	6	0.33 (0.07–0.60)	9	0.47 (0.17–0.78)	12	0.81 (0.35–1.27)	11	0.82 (0.34–1.31)	10	0.81 (0.31–1.31)	30	1.87 (1.21–2.53)	7.35	<.0001
40–59	0	0.00	13	0.92 (0.42–1.42)	12	0.83 (0.36–1.29)	8	0.47 (0.15–0.80)	15	0.75 (0.37–1.13)	21	0.97 (0.56–1.38)	21	0.94 (0.54–1.34)	22	0.95 (0.55–1.34)	78	2.50 (1.96–3.05)	4.51	<.0001
60–100	0	0.00	8	1.19 (0.37–2.01)	8	1.19 (0.37–2.01)	11	1.35 (0.56–2.14)	16	1.66 (0.86–2.47)	22	1.99 (1.17–2.81)	26	2.14 (1.33–2.96)	22	1.59 (0.93–2.25)	46	2.38 (1.70–3.05)	2.18	0.0296

^a^Adjusted by the direct method to the year 2010 Census population using the age groups 18–39 years, 40–59 years, and 60–100 years

*CHNS* China Health and Nutrition Survey

The results of the trends in all obesity-related indicators are expressed as annual changes in *ORs* and displayed in Table 6. For all indicators, there were significant increases in the *ORs* in the total sample and both men and women (all *P* < 0.0001). Compared to women, higher *ORs* in all indicators were observed in men with the exception of grade 2 obesity.

**Table 6:** Estimated annual increase in the odds of obesity profiles prevalence among the Chinese adults by sex and age from the CHNS: 1989–2011

Indicators	Overweight		Obesity		Abdominal obesity		Grade 1 obesity		Grade 2 obesity		Grade 2 and 3 combined obesity	
	*OR*(95%*CI*)	*P*	*OR*(95%*CI*)	*P*	*OR*(95%*CI*)	*P*	*OR*(95%*CI*)	*P*	*OR*(95%*CI*)	*P*	*OR*(95%*CI*)	*P*
Total	1.041(1.039–1.043)	<.0001	1.074(1.070–1.078)	<.0001	1.073(1.070–1.076)	<.0001	1.07(1.066–1.074)	<.0001	1.087(1.073–1.102)	<.0001	1.108(1.094–1.123)	<.0001
Men												
Overall	1.055(1.052–1.058)	<.0001	1.087(1.081–1.093)	<.0001	1.089(1.083–1.094)	<.0001	1.082(1.075–1.088)	<.0001	1.117(1.089–1.147)	<.0001	1.148(1.120–1.178)	<.0001
Age												
18–39	1.056(1.050–1.061)	<.0001	1.125(1.113–1.137)	<.0001	1.104(1.093–1.114)	<.0001	1.118(1.106–1.130)	<.0001	1.195(1.133–1.261)	<.0001	1.223(1.159–1.290)	<.0001
40–59	1.045(1.040–1.050)	<.0001	1.077(1.068–1.087)	<.0001	1.085(1.078–1.093)	<.0001	1.072(1.062–1.082)	<.0001	1.099(1.056–1.143)	<.0001	1.147(1.101–1.194)	<.0001
60–100	1.046(1.038–1.054)	<.0001	1.028(1.016–1.041)	<.0001	1.048(1.039–1.058)	<.0001	1.025(1.011–1.038)	0.0002	1.045(0.997–1.096)	0.0658	1.062(1.017–1.109)	0.0061
Women												
Overall	1.030(1.027–1.033)	<.0001	1.065(1.060–1.070)	<.0001	1.068(1.064–1.072)	<.0001	1.061(1.055–1.066)	<.0001	1.074(1.056–1.091)	<.0001	1.09(1.073–1.107)	<.0001
Age												
18–39	1.013(1.008–1.018)	<.0001	1.065(1.054–1.076)	<.0001	1.060(1.052–1.067)	<.0001	1.055(1.043–1.066)	<.0001	1.136(1.092–1.182)	<.0001	1.164(1.120–1.209)	<.0001
40–59	1.025(1.020–1.029)	<.0001	1.047(1.040–1.055)	<.0001	1.052(1.046–1.058)	<.0001	1.044(1.036–1.052)	<.0001	1.052(1.026–1.079)	<.0001	1.070(1.045–1.097)	<.0001
60–100	1.028(1.021–1.035)	<.0001	1.042(1.032–1.052)	<.0001	1.054(1.045–1.062)	<.0001	1.042(1.031–1.053)	<.0001	1.021(0.994–1.048)	0.1354	1.032(1.006–1.059)	0.0146

*CHNS* China Health and Nutrition Survey

## Discussion

The present study showed that there were significant increases in the age-adjusted prevalence of overweight and general obesity defined by BMI as well as abdominal obesity defined by WC in Chinese adults in the past 22 years. Compared to women, the changes in BMI and WC were particularly pronounced in men. Moreover, the age-adjusted prevalence of overweight in men was greater than that in women. However, the age-adjusted prevalence of abdominal obesity was reversed. Notably, according to the annual *ORs,* the increases in the prevalence of all indicators in men were greater than those in women, with the exception of grade 2 obesity. The annual *ORs* of general obesity, abdominal obesity, and grade 1 obesity decreased significantly with age in men.

In this study, dramatic increases in the prevalence of overweight, general obesity, and abdominal obesity were observed among Chinese adults from 1989 to 2011. The increases occurred in almost all studied sex and age groups, which was accordance with the previous studies [17, 27, 28] . Moreover, the increasing trends in all indicators appeared to continue but not slow or level off. If no effective intervention is implemented to control the prevalence of obesity, China will follow in the footsteps of the U.S., which will lead to an obesity crisis [29, 30]. A previous study reported that the Chinese diet was shifting toward a Westernized diet, as characterized by the proliferation of fast food chains since the late 1980s [31]. As a result, the consumption of animal food and edible oil has dramatically increased; in contrast, the intake of cereals and starchy roots has declined [15]. Therefore, the obesity epidemic in China is attributed to the increasing availability of food, the lack of physical activity, and the Westernization of the dietary pattern.

WC is a simple and effective measure of abdominal obesity and has often been shown to be a strong predictor of an increased risk of hypertension, diabetes, dyslipidemia, metabolic syndrome, and coronary heart disease, independent of BMI [32, 33]. In this study, the age-adjusted prevalence of abdominal obesity defined by WC considerably increased from 1989 to 2011, especially in women, which was in line with the previous study [27]. However, a previous study reported that the distribution of higher WC greatly increased from 1993 to 2009 in men [17]. In 2011, the age-adjusted prevalence of abdominal obesity in women was 50.75%. Note that the prevalence of abdominal obesity in the 40–59 years old and 60–100 years old groups were 61.11 and 68.20% in 2011, respectively. Therefore, the high prevalence of abdominal obesity poses a serious public health challenge in China.

According to the annual *ORs*, there were significant increases in the prevalence of all obesity-related indicators. Compared to women, there were more rapid increases in all indicators except grade 2 obesity in men. There were significant differences in the increasing rates of general obesity, abdominal obesity, and grade 1 obesity across the three age groups in men. And the annual *ORs* decreased significantly with age. Therefore, the obesity population is trending toward a higher proportion of males and younger individuals in China, which should be examined in a well-designed study in the future.

In this study, it was found that the prevalence of all obesity-related indicators increased more rapidly in men than that in women, which was in line with the findings of previous studies [14, 17, 28, 34]. The sex disparity might be explained by sociocultural, socioeconomic, behavioral, and genetic factors. First, obesogenic environmental changes resulting in high calorie intake might have contributed to male dominance in obesity increases. Furthermore, sex hormone responses to obesogenic environmental changes need to be considered [35]. Second, the dietary and physical activity behavioral differences between men and women might partly explain the sex disparity [16]. Third, body image dissatisfaction is more prevalent in women in China [36, 37]. The Chinese 2005 NYRBS (National Youth Risk Behavior Surveillance) showed that 23.6% of girls and 9.1% boys tried to lose weight by restricting their diets [38]. This might explain why the prevalence of obesity increased more slowly in women. The prevalence of abdominal obesity in women was higher than that in men, which might be attributed to hormonal levels. When women experience from menopause, estrogen declines rapidly, and follicle stimulating hormone increases. As a result, the accumulation of visceral fat is exacerbated [39]. Therefore, the prevalence of abdominal obesity would increase more rapidly in women.

### The strengths and limitations

Data were obtained from the nationally representative CHNS. Thus, the findings of this study present the true and dynamic description of obesity-related variables in China. Because of the differences in ethnicities and dietary patterns among different countries, the prevalence and extent of obesity vary. Specific cut-offs of BMI should be used to define overweight and obesity in each country. In this study, according to the WHO recommendations for Chinese people, ethnicity-based cut-offs for BMI were used to define overweight and obesity. Therefore, the results of this study provided accurate and realistic estimations of the prevalence of overweight, general obesity, and abdominal obesity in China. However, the limitations of this study should be stated. Since the measurement of WC in the CHNS began in 1993, the prevalence of abdominal obesity and the distribution of WC were not reported in 1989 or 1991. The study population focused on children and adults aged ≤45 years old in 1989, which led to no result presented in the 60–100 years old group.

## Conclusions

The prevalence of overweight, general obesity, and abdominal obesity increased significantly among Chinese adults from 1989 to 2011. The median BMI and WC increased rapidly over the 22 years. The annual *ORs* indicated that the increases in the prevalence of overweight, general obesity, and abdominal obesity in men were more rapid than those in women. Therefore, the obesity population is trending toward a higher proportion of males and younger individuals in China.

## Data Availability

The datasets generated and/or analyzed during the current study are available in the web: https://www.cpc.unc.edu/projects/china.

## Abbreviations

BMI: Body mass index
CCDC: Center for Disease Control and Prevention
CHNS: China Health Nutrition Survey
*CI*: Confident interval
NYRBS: National Youth Risk Behavior Surveillance
*ORs*: Odds ratios
WC: Waist circumference
